# Experience shapes accuracy in territorial decision-making in a poison frog

**DOI:** 10.1098/rsbl.2020.0094

**Published:** 2020-05-13

**Authors:** Ria Sonnleitner, Max Ringler, Matthias-Claudio Loretto, Eva Ringler

**Affiliations:** 1 Department of Evolutionary Biology, Unit of Integrative Zoology, University of Vienna, Althanstraße 14, 1090 Vienna, Austria; 2 Department of Behavioural and Cognitive Biology, University of Vienna, Althanstraße 14, 1090 Vienna, Austria; 3 Department of Migration, Max Planck Institute of Animal Behavior, Am Obstberg 1, 78315 Radolfzell, Germany; 4 Department of Biology, University of Konstanz, Universitätsstraße 10, 78464 Konstanz, Germany; 5 Messerli Research Institute, University of Veterinary Medicine Vienna, University of Vienna, Veterinaerplatz 1, 1210 Vienna, Austria

**Keywords:** territoriality, aggression, experience, accuracy, poison frogs

## Abstract

The trade-off between speed and accuracy affects many behavioural processes like predator avoidance, foraging and nest-site selection, but little is known about this trade-off relative to territorial behaviour. Some poison frogs are highly territorial and fiercely repel calling male intruders. However, attacks need to be conducted cautiously, as they are energetically costly and bear the risk of own injury or accidentally targeting the wrong individual. In this study, we investigated the speed–accuracy trade-off in the context of male territoriality during the breeding season in the brilliant-thighed poison frog, *Allobates femoralis*. In our experiment, we presented the call of an invisible ‘threatening’ intruder together with a visible ‘non-threatening’ intruder, using acoustic playback and a frog model, respectively. Contrary to our prediction, neither reaction time nor approach speed of the tested frogs determined the likelihood of erroneous attacks. However, younger individuals were more likely to attack the non-threatening model than older ones, suggesting that experience plays an essential role in identifying and distinguishing rivalling individuals in a territorial context.

## 1. Background

Animals that spend more time accumulating information before a behavioural response face lower error rates than faster-acting individuals [1,2]. This so-called speed–accuracy trade-off implies that speed and accuracy in decision-making cannot be maximized simultaneously; for information about the neural basis thereof see [3,4]. Many behavioural patterns are affected by this trade-off; for example, the avoidance of hidden predators which has been shown in mammals [5] and bumblebees [6], the foraging strategies examined in pollinating insects [7,8], and the nest-site selection observed in house-hunting ant colonies [9]. However, very little is known about the speed–accuracy trade-off affecting territorial behaviour.

Territory defence is widespread across amphibians and particularly prominent in all Neotropical poison frogs (Dendrobatidae: Aromobatinae *sensu* [10]) that have been studied [11], for instance in *Allobates femoralis*, a small species that is widespread across Amazonia and the Guiana Shield [12]. The reproductive success of male *A. femoralis* appears to be influenced by their ability to hold multi-purpose long-term territories in which pair-formation, courtship, mating and oviposition take place [13–16]. During the reproductive season males establish and claim territories by calling from elevated perches on the forest floor [13,15,17]. Other calling males are not tolerated within a territory and dislodged by aggressive behaviour, such as antiphonal calling, direct phonotactic approach or chasing and attacking the intruder [14,18–20]. The level of territorial aggression depends on the received sound pressure level (SPL) of a calling intruder, as well as on its perceived distance [21]. SPLs greater than 56 dB evoke orientation towards the sound source followed by antiphonal calling (56–68 dB) or by an approach towards the sound source (greater than 68 dB) [17,19]. Previous studies using acoustic playbacks and robotic frog models (FMs) have shown that physical combat is only elicited reliably when the acoustic and visual signals, such as vocal sac pulsations [18] or body movement [22] of the model, are presented concurrently. During previous fieldwork, we have occasionally observed males attacking nearby, non-calling males or females (E.R. and M.R., personal observations), prompting the question whether accuracy in the context of territorial defence suffers from quicker response and higher approach speed in *A. femoralis.* Therefore, we conducted an experiment to simulate an invisible, threatening intruder, using a playback call, together with presenting a visible, non-threatening intruder, using a moving, robotic FM without visual cues of calling (i.e. pulsating vocal sac). We expected males with faster decision time and/or approach speed to conduct more unwarranted attacks on the FM.

## 2. Material and Methods

### (a) Study site

The study was conducted in lowland tropical rainforest in an experimental *A. femoralis* population that had been installed on a small (approx. 5 ha) island [23] in the River Arataye in the Nouragues Nature Reserve in French Guiana, close to the ‘Saut-Pararé’ field camp (4°02′ N 52°41′ W) of the Nouragues Ecological Research Station. The study was carried out February–March in 2017 and 2019, during the reproductive period of our study species, which coincides with the local rainy season. All experiments were conducted during the two peaks of *A. femoralis* calling activity (07.00–12.00 h, 14.00–19.00 h).

### (b) Experimental design

**Figure 1.**
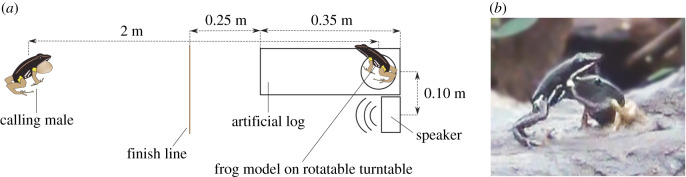
(*a*) Experimental setup (not to scale), showing the distances between the rotating frog model (FM), the focal male and the loudspeaker. (*b*) Extracted picture from video footage showing an *A. femoralis* male attacking the FM.

To investigate whether a fast initial decision time and/or approach speed reduce attack accuracy, we conducted a bimodal intrusion experiment (figure 1*a*), using the FM as the visual component and a standardized advertisement playback call as the acoustic cue.

We used a FM from previous studies [18,24], integrated into an artificial log (0.35 × 0.12 × 0.12 m) made from epoxy resin. The FM was made from silicone rubber, painted like an adult *A. femoralis*, and fitted on a turntable that was rotated with a servo motor (Graupner Modellbau) at an angular velocity of approximately 1.3 rad s^−1^ between 90° left and right of the axis between focal male and FM. In our study, the FM's vocal cavity was filled with modelling clay to mimic a female or a non-calling male. After locating a calling male, the FM was placed at an estimated distance of 2 m inside the focal male's territory.

We broadcast the acoustic stimulus using a portable speaker with an integrated audio player (Creative MUVO 2c), placed 10 cm left or right (alternated between trials) beside the FM, and oriented towards the calling focal male. We chose this lateral positioning over placing the speaker behind the FM to avoid guiding frogs directly into the FM during their phonotactic approach, which usually follows a straight line. The distance between FM and loudspeaker corresponds to an angle of 2.9° at a distance of 2 m, which allowed reliable integration of both signals in previous studies [24]. Preliminary tests with larger separation distances (50 cm) resulted in very few attacks on the FM and were thus excluded from the present experiment. For the acoustic stimulus, we used the artificial ‘standard call’ *sensu* Ursprung *et al*. [20], which is based on recordings by Gasser *et al*. [25]; for a detailed description see [21]. We calibrated the speaker every day to broadcast the playback with the same volume (75 dB measured at 2 m distance) in all trials.

To obtain standardized measures of approach speed, we measured the time from the first jump until the focal males approached to within 0.25 m of the artificial log (figure 1). For this, we designated a ‘finish line’ by positioning small twigs on the forest floor. Frog behaviour during the trials was recorded using a voice recorder (Sony ICD-PX333), with the observer placed approximately 2 m to the side of the setup. Each trial started as soon as the loudspeaker and the turntable were activated and lasted for 25 10-call bouts (total 402.4 s). No individual was tested twice in our experiment.

We measured the exact initial distance between the male and the FM (*X̅* ± s.d. = 2.23 ± 0.30 m) with a laser rangefinder (Bosch DLE 50) at the end of each trial. Additionally, we measured the ambient temperature (*X̅* ± s.d. = 25.9 ± 0.8°C) using a digital hygro-/thermometer (Greisinger GFTH 95). Relative humidity was always at 100%, and not further considered in analyses. After each trial, we measured the SPL of the playback signal at the initial position of the focal male using a sound level meter (Voltcraft SL-100). Owing to the structural complexity of the forest floor, the received SPL of the playback signal differed among trials between 64.5 and 76.9 dB. We determined male identity and age from our long-term monitoring of the closed island population with a sampling coverage of greater than 90%, allowing differentiation between young (i.e. new encounters in a given year) and old individuals (i.e. recaptures from previous years). We used dorsal pictures on a millimetre-grid to measure snout–urostyle length (SUL; *X̅* ± s.d. = 28.6 ± 1.2 mm) using the open source program ‘Fiji’ [26].

In total, we tested 51 males but excluded four individuals that did not move at all and eight individuals that did not reach the finish line. Hence, our final dataset of 39 males included 22 ‘old’ and 17 ‘young’ individuals. Nineteen trials were conducted in 2017, and 20 trials in 2019.

### (c) Data analysis

We used the software Solomon Coder [27] to transcribe observational recordings into behavioural timetables. The ‘initial decision time’ was defined as the time measured from the beginning of the trial (i.e. start of the playback and activation of the turntable) until the male performed its first jump. The ‘approach speed’ was calculated by dividing the distance between the tested male and the finish line (which was determined from the exact initial distance between the male and the FM) by the time it took the individual from its first jump to reach the finish line.

To investigate a possible speed–accuracy trade-off, and the possible influence of environmental (°C, SPL, test year) or individual (age, SUL) factors on the probability of attack, we created generalized linear models (glms) with binomial error structure. We used attack (yes/no) as the response variable and approach speed, initial decision time, °C, SPL, age, year and SUL as predictors (see the electronic supplementary material).

There was no multicollinearity between the predictors (variance inflation factor, VIF ≤ 2.1 for all predictor variables). We standardized all variables by centring and dividing by 2 s.d. [28] to make their effect sizes comparable independent from their scale. For model selection, we followed an information-theoretic approach [29] and created a set of candidate models with all possible combinations of the predictor variables from the respective full model. We ranked the models based on Akaike's second order information criterion (AICc) [30] and selected the subset of best models within ΔAICc ≤ 6 [31] to calculate model-averaged coefficients. All statistical analyses were done in R [32] using the packages lme4 [33] and car [34] for calculating the VIF, and MuMIn [35] for model averaging.

## 3. Results

Overall, seven out of 39 individuals that approached the playback also attacked the FM, where six of those were first-year adults (i.e. ‘young’ individuals). No difference in attack behaviour was observed between the two sampling years. The estimated model-averaged coefficient for age had the highest relative importance and a very strong, negative effect on the probability to attack (estimate = −2.44, s.e. = 1.5, RI = 0.91; table 1). SPL also had a high relative importance, with a strong positive effect on the probability to attack. This means that older individuals were less likely to attack the FM, and the probability of an attack increased with the SPL of the playback signal at the initial location of the tested male. The differences in attack likelihood between young and older individuals could not be attributed to differences in SPLs, which were not significantly different between age classes (*t*-test: *N* = 29, *t* = 0.975, *p* = 0.337). Five males approached the artificial log although SPL was below the previously proposed threshold of 68 dB to elicit phonotactic response [17] (with 64.5 dB SPL as the lowest).

**Table 1.** Model-averaged coefficients including standard errors (s.e.) and relative importance (RI) to explain the probability of an attack.

	estimate	s.e.	RI
intercept	−2.64	1.03	—
age	−2.44	1.50	0.91
sound pressure level (SPL)	2.10	1.89	0.76
initial decision time	−2.28	3.03	0.55
approach speed	0.29	0.79	0.27
snout–urostyle length (SUL)	−0.30	0.80	0.28
year of data sampling	0.35	0.96	0.26
temperature (°C)	0.28	0.88	0.25

Initial decision time had a strong negative effect on the probability to attack, but a much smaller relative importance and a comparably large s.e. and is, therefore, hard to interpret. Approach speed, SUL, year and °C had a low relative importance and small effect sizes, and are, therefore, least likely to have affected attack behaviour.

## 4. Discussion

Contrary to our prediction, neither initial decision time nor approach speed affected attack accuracy in *A. femoralis* males. Our results rather suggest a fundamental role of experience in territorial decision-making, as age had the largest influence on attack probability.

The finding that older individuals were less likely than young ones to attack the FM might reflect a difference in experience. It is known from several animal species that experience plays a major role in many contexts, such as hunting [36–38], foraging [39,40] or mating [41–43]. A considerable impact of age and/or experience on territory defence behaviour has already been shown in birds [44,45] and insects [46,47]. We assume that older *A. femoralis* individuals are more experienced and therefore might already have learned to visually distinguish between calling and non-calling males, as well as between males and females. Therefore, older frogs might be better in discriminating between threatening intruders and non-threatening individuals. The relatively low attack rate (7 out of 39) in our study is in line with the general assumption that fighting is energetically costly and involves the risk of getting injured. Furthermore, attacking a female by mistake might result in losing a valuable mating opportunity. Therefore, any attack needs to be well considered. The idea that frogs might need to learn to discriminate between sexes and threat potential of intruders is supported by our field observations where males have attacked females sojourning in their territory while another male is calling close by (E.R., M.R. and R.S., personal observations), and males performing courtship calls towards non-calling males (E.R. and M.R., personal observations). Theoretically, the lower attack rate in older individuals could also be caused by effects of senescence (e.g. owing to older individuals being less likely to win fights). We, however, do not think that this is the case in our study, as we did not observe any differences in their phonotactic response; the overall ‘readiness to fight’ was equal across age groups.

Although attacking a non-threatening intruder, if it is a female, might cause the loss of a mating opportunity, young individuals still might benefit from this behaviour. We assume that such quick response might support establishment of a territory in the first place, which in the long run likely is more important than a single lost mating, as it ensures reproductive success over weeks to months [15].

Attacks were also motivated by higher SPLs received by the tested males. We assume that the intruder's SPL provides information about its threat potential—a closer [21] and/or larger intruder represents a more imminent threat than a distant caller, which might lower the territory owner's accuracy in decision-making. Our results show no effect of body size nor of ambient temperature on attack likelihood, likely because we only tested adult territorial individuals with little variation in body size (SUL: *X̅* ± s.d. = 28.6 ± 1.2 mm) and across a relatively small range of ambient temperatures (temperature: *X̅* ± s.d. = 25.87 ± 0.79°C), as trials were only conducted under conditions when frogs showed normal levels of calling activity.

We conclude that in *A. femoralis* accuracy in territorial decision-making is shaped by lifetime experience. Particularly in species without strong sexual dimorphism, as in *A. femoralis*, individuals might need longer to learn to discriminate between males and females, using all available cues (cf. [18,48]). Further experiments are needed to investigate which factors motivate adequate male responses to males and females in this monomorphic species.

## Supplementary Material

Dataset

## Data accessibility

A data file can be found in the electronic supplementary materials.
